# Electromyographic Analysis of the Support Scale in Gymnastics and Its Related Preconditioning Strengthening Exercises

**DOI:** 10.1519/JSC.0000000000005074

**Published:** 2025-02-26

**Authors:** Giuseppe Rosaci, Federico Nigro, Matteo Cortesi, Simone Ciacci, Sandro Bartolomei, Silvia Fantozzi

**Affiliations:** 1 Department for Life Quality Studies, University of Bologna, Bologna, Italy; and; 2 Department of Electrical Energy and Information Engineering, University of Bologna, Bologna, Italy

**Keywords:** surface electromyography, isometric contraction, muscle synergy, planche

## Abstract

Rosaci, G, Nigro, F, Cortesi, M, Ciacci, S, Bartolomei, S, and Fantozzi, S. Electromyographic analysis of the planche in gymnastics and its related preconditioning strengthening exercises. *J Strength Cond Res* 39(6): 680–686, 2025—The support scale (SS) is a common upper body skill in gymnastics. Athletes typically introduce preconditioning strengthening exercises (PSEs) in their training routines to prepare this strength element. Despite the popularity of these exercises, their effectiveness in reproducing the muscle involvements of the SS is unknown. The aim of this study is to compare the muscular excitations, coactivation indices, and synergies between the SS and its related PSEs. Seven high-level ring specialist gymnasts (age: 23.9 ± 4.0 years, height: 165.9 ± 2.8 cm, body mass: 65.6 ± 3.1 kg, experience: 13.0 ± 3.0 years) performed the SS and 5 PSEs. Electromyographic activities of the pectoralis major (clavicular part), latissimus dorsi, triceps (long head), infraspinatus, trapezium (transverse), serratus anterior, biceps brachii, and anterior deltoid were recorded, and muscle synergies were studied. Large differences between the SS and its 5 PSEs were detected in the muscle activity of trapezius (*F* = 8.937; *p* < 0.001; η^2^ = 0.641), pectoral (*F* = 5.235; *p* = 0.002; η^2^ = 0.512), and biceps muscles (*F* = 10.359; *p* < 0.001; η^2^ = 0.674). The coactivation was different in the biceps/triceps ratio (*F* = 5.980; *p* < 0.001; η^2^ = 0.545) and the serratus/trapezium ratio (*F* = 8.043; *p* < 0.001; η^2^ = 0.617). The analysis of muscle synergies showed different simultaneous muscular activation in the PSEs compared with the SS. Thus, the results of this study provide evidence for a different use of PSEs in the training routine to improve SS performance.

## Introduction

**Figure 1.**
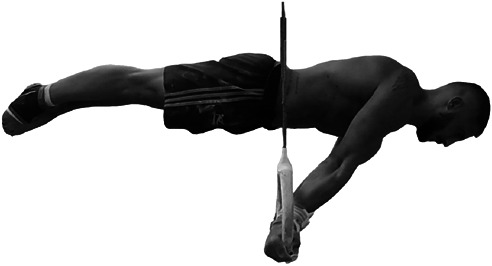
The support scale executed on official competition rings.

The rings represent 1 of the 6 apparatuses involved in the male artistic gymnastics Olympic program. An analysis of the World Championships finals from 2017 to 2019 revealed that the 62.3% of the elements executed in the rings routine included hold elements (23). These data demonstrated the key role of the static elements for the final competitive score on the rings (23). The swallow, the iron cross, and the support scale (SS) (also called “planche”) (19) are elements frequently performed in rings competitions and often combined with swing elements. More specifically, the SS element is frequently used by athletes to improve upper body strength and body control (20). This exercise involves assuming and holding a horizontal body position, with arms fully extended above the rings (Figure 1).

To strengthen the SS element, athletes commonly incorporate exercises that use pulley systems, dumbbells, barbells, or loop bands into their training routine. These exercises, which aim to replicate the muscle excitation and actions of the SS element, are defined as preconditioning strengthening exercises (PSEs) (19). Previous studies have investigated the muscle excitation of the shoulder and trunk muscles during the PSEs in comparison with the swallow and iron cross elements performed on competitive rings, using bipolar surface electromyography (EMG). Bernasconi et al. (7) reported that the pulley system with counterweight, adopted to train the swallow and to replicate its holding element, induced a lower muscle excitation of the pectoralis major muscle than the main element. In contrast, barbell and dumbbell PSEs resulted in similar muscle coordination during shoulder flexion, compared with swallow. On the contrary, in these exercises, the coactivation index of the biceps brachii and triceps muscles and the contribution of the serratus anterior in stabilizing the scapulae were altered compared with the swallow. In addition, according to Bernasconi et al. (6) and Carrara et al. (11), the use of “herdos” (special supports that decrease the arm's lever) during iron cross training reduces muscular stress, shoulder asymmetry, and additional movements from the 90° angle in the iron cross performance (6,11). Therefore, considering the intimate connection between exercises and technical elements, the PSEs may play a crucial role in improving performance and optimizing training.

Muscle synergy analysis has been used to study the coordination strategies of the elbow and shoulder muscles during isometric strength tasks (5,9,27). Consequently, the study of muscular excitation, coactivation indices, and synergies in gymnastics movements may help trainers and researchers to identify the characteristics of the technical elements of gymnastics and to establish which exercises should be included in the training routine in relation to the main focus of the workout. To the best of our knowledge, no studies to date analyzed the muscular activity during the SS and its related PSEs. Therefore, the aim of this study was to analyze the muscular excitation, coactivation indices, and synergies of the SS element, and to determine the specificity of each PSEs for optimal use in training. The authors hypothesized that different muscle activations and synergies may be found in some PSEs compared with SS.

## Methods

### Experimental Approach to the Problem

This investigation was an observational comparative research study. Athletes participating in the study were tested during a single morning visit to their own clubs, where standard competitive rings were installed. Participants were asked to abstain from training for the 24 hours before the study. Athletes were asked to perform the SS and its 5 PSEs, typically used in each athlete's training routine, in a randomized order. For this purpose, Research Randomizer (Urbaniak GC. & Plous S.; Version 4.0) web service was used. The settings used were as follows: 1 set with 6 numbers, each ranging from 1 to 6. In addition, each number was kept unique, and no sorting or marking options were applied to the results. During the SS element and its 5 PSEs, muscle excitation was recorded using EMG. In addition, the coactivations and synergies between the different muscles were calculated (15,21) to study the differences between exercises.

### Subjects

Seven national-level gymnasts, specializing in the rings event (age 23.9 ± 4.0 years, height 165.9 ± 2.8 cm, and body mass 65.6 ± 3.1 kg), participated in this study. All participants were highly trained (year of experience: 13.0 ± 3.0 years, training time per week: 21.2 ± 1.9 hours, score 14.007 ± 0.538) and free from musculoskeletal injuries in the 6 months before the study. The inclusion criteria were the ability to perform the SS element on regular rings with horizontal shoulders, hips, and feet alignment. Before the beginning of the study, each participant signed an informed consent, and the study was approved by the local Bioethics Committee of the University of Bologna (n. 0139105; June 20, 2022).

### Procedures

Participants performed a standardized general warm-up as previously described by Bartolomei et al. (3), followed by 5 minutes of self-administered specific warm-up selected by each participant. After the warm-up, each athlete performed the SS and 5 different PSEs once. If the first attempt did not conform to the gymnastics scoring system and posture criteria (straight arms, shoulders flexed until the hands reach the vertical plane of the hips and scapular abduction) (13), a second attempt was required after 5 minutes. The criteria were assessed by experienced gymnastics coaches (>10 years of experience) during the execution of each exercise. The PSEs performed were SS at cable pulley (SS_P_), resistance bands in prone decubitus (RPD), SS with supported feet (SS_SF_), dumbbells in supine decubitus (DSD), and dumbbells in standing position (DSP). All the exercises are depicted and described in Table 1. The same pulley system (G-Force Training System, Torino, Italy) was used for the SS_P_ exercise. Despite no studies to date investigated these exercises, similar PSEs were described in previous investigations (19,20).

**Table 1:** Preconditioning strengthening exercises (PSEs) commonly used by gymnasts to improve the support scale performance on the competition rings.

Preconditioning strengthening exercises (PSEs) for the support scale	
Image	Descriptions
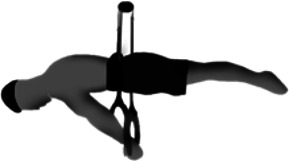	**Support scale at cable pulley (SS**_**P**_**)** The athlete performs the SS using a cables system with 2 pulleys. The one end of each cable is connected to a ring while at the other end the cable is connected to the athlete's body by a specific belt
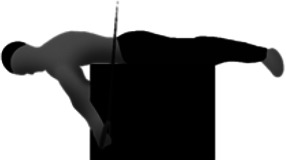	**Resistance band in prone decubitus (RPD)** The athlete lays in prone position, with the arm straight at 45° of abduction. The athlete grasps and pulls 2 elastic bands until his hands are just below the line of the pelvic bones
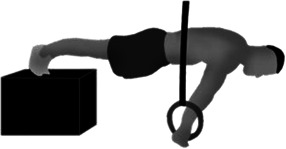	**Support scale with supported feet (SS**_**SF**_**)** The SS_SF_ consists in the support scale performed on regular rings but with the feet supported by a step, at the same height of the shoulders line and with the hands perpendicular to the pelvic bones
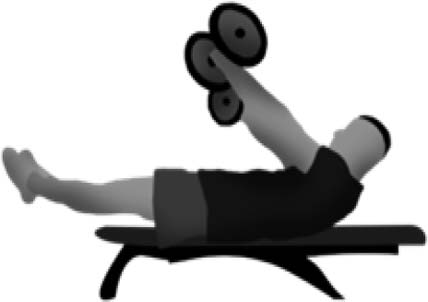	**Dumbbells in supine decubitus (DSD)** In DSD the athlete lays in supine position, with the arm straight at the side of the pelvis and abducted at 45°. The athlete is requested to lift 2 dumbbells until the hands are just above the line of the pelvic bones
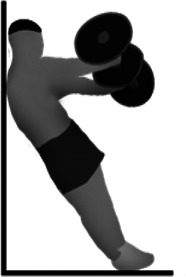	**Dumbbells in standing position (DSP)** The athlete is required to lift the dumbbells with straight arms and a supine grip, until the hands are parallel to the ground. The athlete performs this movement from a standing position with the shoulders in contact with the wall and the trunk at 45° to the ground

The exercises were separated by a recovery time of 5 minutes. In each holding exercise, the isometric contraction was maintained for 2 seconds (SS, SS_P_, SS_SF_). The dynamic exercises (RPD, DSD, DSP) were performed with a load that allowed a maximum of 3 repetitions. Participants were instructed to hold the weight for 2 seconds in each repetition of each exercise. This setting was chosen to simulate typical gymnastics training conditions (7). Specifically, in the RPD exercise, the load was modulated using elastic bands, whereas in the DSD and DSP exercises, the whole mass of the dumbbells corresponded to 44.5 ± 4.5% and 36.7 ± 4.9% of the athlete's body mass, respectively.

#### Instruments and Data Analysis

The wireless EMG system (Cometa systems Inc., Milano, Italy; resolution16-bit, gain 1,000, impedance 10^5^ Ω) was used to measure muscles signals. The skin area under the EMG electrodes was shaved and cleaned with ethyl alcohol to reduce impedance. The signal, acquired at a frequency of 2000 Hz, was detected by 2 Ag/AgC1 electrodes (30 × 24 mm; H124SG, CardinalHealth, Kendall, United Kingdom) with an interelectrode distance of 2 cm. Electrodes were placed on 8 trunk and shoulder muscles on the left side of the athlete's body: biceps brachii (BIC), transverse trapezius (TRA), anterior deltoid (DEL), and triceps long head (TRI), following European SENIAM procedure (17). Pectoralis major clavicular head (PEC), serratus anterior (SER), infraspinatus (INF), and latissimus dorsi (DOR) were recorded based on the literature (12,25,26,28). Crosstalk was measured by selective movement for each muscle prior the acquisition. The signals were recorded for 2 seconds during the execution of each PSEs and were used for subsequent data elaborations. Initially, EMG signals underwent bandpass filtering between 10 and 500 Hz using a second-order zero-lag IIR Butterworth filter. The root mean square (RMS) of the EMG signals was then calculated with a 25 milliseconds window for each muscle in each exercise. The ratio between the RMS of the PSEs and the SS, expressed in percentage, was used to calculate the muscular activation of the PSEs relative to the main element. A coactivation index, based on the antagonist/agonist muscles ratio (21), was calculated for PEC/DOR, BIC/TRI, SER/TRA, and DEL/INF as

$$\text{Co}-\text{activation}\;\text{Index}=\frac{{\text{RMS}}_{\text{ANT}}}{{\text{RMS}}_{\text{AG}}+{\text{RMS}}_{\text{ANT}}}\times 100.$$

Muscle synergies were extracted for each muscle and exercise as described in Ghislieri et al. (15,16). After the bandpass filtering previously described, EMG signals underwent a sequence of processing steps as follows: high-pass filtering (eighth order zero-lag IIR Butterworth digital filter, cutoff frequency of 35 Hz), rectification, and low-pass filtering (fourth order zero-lag IIR Butterworth digital filter, cutoff frequency of 12 Hz). Electromyography envelope was then amplitude normalized to the maximum value observed across all acquired muscle signals for each participant. To extract a set of N time-invariant muscle synergies that minimize the reconstruction error, the Non-Negative Matrix Factorization algorithm was used (12,14). The “nnmf” MATLAB function was run several times changing the number of muscle synergies from 1 to 8 using the input parameters optimized in previous works: multiplicative update, 1e^−6^ function tolerance, 50 replicates, and 1,000 iterations (max.) (12,14). For each number of muscle synergies, the R^2^ similarity between the original and the reconstructed EMG data was computed. Thus, the number of muscle synergies necessary for accurate reconstruction of the original signals was determined as the point at which the curve of R^2^ against the number of synergies exhibits the most significant change in curvature, often referred to as the “elbow” criterion.

### Statistical Analysis

Shapiro–Wilk test was used to assess the normal distribution of data. Differences in muscle excitation and coactivation indices were calculated using a repeated measure ANOVA. If the ANOVA results were significant, pairwise comparisons between the SS and the 5 PSEs were performed using the Bonferroni's post hoc test. The significant alpha level was set at 0.05 for all analyses. In addition, Cohen's d effect sizes (ES) were calculated to determine the magnitude of differences between interactions: small >0.2, medium >0.6, large >1.20, and very large >2 (18). All statistical analyses were performed using SPSS software (version 28, IBM, Armonk, NY).

## Results

### Support Scale

The EMG analysis of the SS showed values above 1.500 µV in the BIC (1737.7 ± 668.5 µV) and DEL (2043.2 ± 763.1 µV) muscles. Values close to 1.500 µV were detected in SER (1.442.1 ± 443.4 µV), while values for the other muscles were below 1.000 µV (PEC: 907.6 ± 284 µV; INF: 495.6 ± 219.3 µV; TRI: 153.8 ± 54.3 µV; DOR: 95.0 ± 33.5 µV). The coactivation index analysis showed that the highest ratio was detected between SER/TRA (32.7%) and the lowest ratio between BIC/TRI (9%). The PEC/DOR and DEL/INF were 9.9 and 19.9%, respectively. Four synergy patterns were extracted during the SS. The analysis of the muscle synergies during the SS element showed simultaneous activation of PEC and INF in W1; of SER, DEL, and BIC in W2; of DOR and INF in W3; and of INF, TRA, SER, and BIC in W4.

### Comparison with Preconditioning Strengthening Exercises

**Figure 2.**
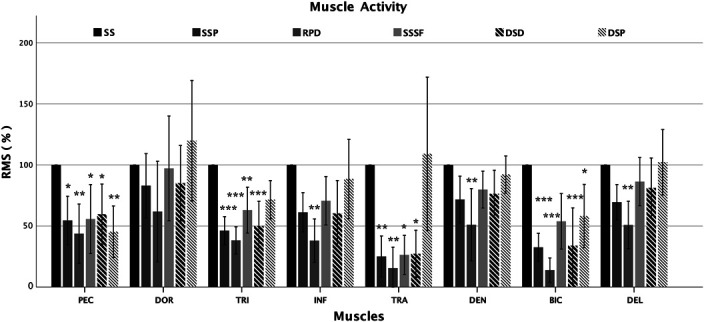
RMS percentage of the PSEs in relation to the main element. Error bars indicate standard deviation. Significances: *p < 0.05; **p < 0.01; ***p < 0.001. SS = support scale; SS_P_ = Support scale at cable pulley; RPD = resistance band in prone decubitus; SS_SF_ = support scale with supported feet; DSD = dumbbells in supine decubitus; DSP = dumbbells in standing position.

**Figure 3.**
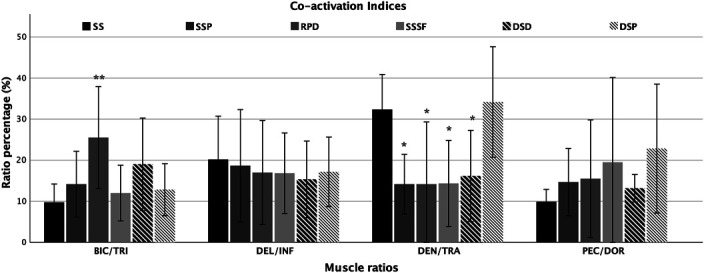
Coactivation indices between agonist/antagonist muscles across the different exercises. Error bars indicate standard deviation. Significances: *p < 0.05; **p < 0.01; ***p < 0.001. SS = support scale; SS_P_ = Support scale at cable pulley; RPD = resistance band in prone decubitus; SS_SF_ = support scale with supported feet; DSD = dumbbells in supine decubitus; DSP = dumbbells in standing position.

The PSEs showed that many differences exist in muscle excitation and coactivation indices in comparison with the SS. Only the DOR muscle did not show significant differences in muscle excitation across the different exercise (*p* > 0.05). On the contrary, the coactivation indices between BIC/TRI, and SER/TRA only, were significantly different compared with the main element (*p* < 0.001). Figures 2 and 3 depict the muscle excitations, coactivation indices, and the statistical differences between the SS and the 5 PSEs.

**Figure 4.**
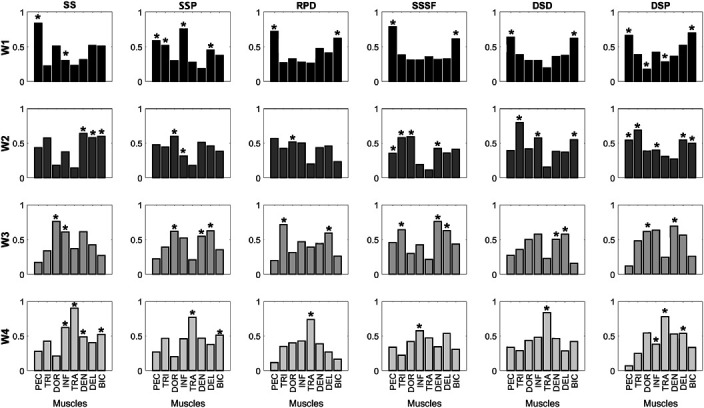
Muscle synergies of support scale (SS), support scale at cable pulley (SS_P_), resistance band in prone decubitus (RPD), support scale with supported feet (SS_SF_), dumbbells in supine decubitus (DSD), and dumbbells in standing position (DSP). *Muscle active in the extracted synergy.

The same number of synergies detected in SS was found in PSEs. However, the contribution of each muscle during the 4 synergies showed different motor recruitments and activations in comparison with the SS (Figure 4).

### Support Scale at Cable Pulley

The EMG muscle analysis of the SS_P_ showed a significantly lower excitation of PEC (−44%; *p* = 0.023; ES = 1.49), TRI (−42%; *p* < 0.001; ES = 2.40), TRA (−70%; *p* = 0.008; ES = 2.09), and BIC (−67%; *p* < 0.001; ES = 2.31) compared with SS. The SER/TRA coactivation ratio was the only index that was significantly lower (16%) compared with the SS (*p* = 0.013; ES = 1.67). The other coactivation indices were similar to SS. The synergies analysis showed a simultaneous muscle activation of PEC, TRI, TRA, and DEL in W1 synergy. Dorsi and INF were active in W2 synergy, while DOR, SER, and DEL muscles were active in the W3 synergy. The W4 synergy showed the activation of TRA and BIC muscles only.

### Resistance Bands in Prone Decubitus

In the RPD exercise, PEC (−56%; *p* = 0.003; ES = 1.81), TRI (−48%; *p* < 0.001; ES = 2.74), INF (−60%; *p* = 0.008; ES = 1.08), SER (−44%; *p* < 0.001; ES = 1.60), and DEL (−49%; *p* = 0.001; ES = 1.29) were significantly lower than in the SS. In addition, the lowest muscle excitation compared with SS was found in BIC (−80%; *p* = 0.001; ES = 3.07) and TRA (−83%; *p* = 0.005; ES = 2.20) muscles. The SER/TRA coactivation index (15.2%) was different from SS (*p* = 0.012; ES = 1.68) and very similar to the SS_P_ results. The BIC/TRI ratio (23.9%) was higher and significantly different compared with the SS (*p* = 0.001; ES = −1.90). The W1 and W2 synergies showed the activity of PEC and BIC, and DOR muscles, respectively. The W3 synergy was characterized by the activation of TRI and DEL muscles, whereas the W4 synergy showed the activation of the TRA muscle only.

### Support Scale with Supported Feet

No significant differences were detected in the BIC muscle excitation (53.8%) between SS_SF_ and SS (*p* = 0.072; ES = 1.43). The analysis of the excitation of the other muscles showed significantly lower RMS values for TRA (−74%; *p* = 0.017; ES = 1.93), PEC (−45%; *p* = 0.026; ES = 1.47), and TRI (−37%; *p* = 0.010; ES = 1.62) than for SS. The SER/TRA coactivation index (14.3%) was significantly different compared with the SS (*p* = 0.014; ES = 1.66). The BIC/TRI coactivation index (12.0%) was the closest to BIC/TRI index of the SS element (9.0%). The synergies analysis highlighted the activation of PEC and BIC in W1 and the activation of PEC, TRI, DOR, and SER in W2. The W3 synergy showed the activity of TRI, SER, and DEL, while the last synergy only showed the activation of the INF muscle.

### Dumbbells in Supine Decubitus

As detected in the early mentioned exercises, TRA excitation was significantly lower in DSD (−71%; *p* = 0.026; ES = 1.84) than in SS. Muscle excitation of the PEC (−37%; *p* = 0.018; ES = 1.53), TRI (−42%*; p* < 0.001; ES = 2.24), and BIC (−65%; *p* < 0.001; ES = 2.32) registered in DSD was significantly lower than in the SS element. A significant difference in coactivation indices was detected for SER/TRA ratio only (16.5%, *p* = 0.038; ES = 1.484). The BIC/TRI ratio was higher in DSD (18.0%) than in the SS (9.0%), while the difference was lower for the PEC/DOR ratio (12.5 and 9.9% in DSD and SS, respectively). Muscle synergies revealed a simultaneous activity of PEC and BIC in W1 and an activation of TRI, INF, and BIC in W2. The W3 synergy showed the activation of SER and DEL, while the W4 synergy indicated the activation of TRA only.

### Dumbbells in Standing Position

The DSP exercises showed a lower excitation of PEC (−47%; *p* = 0.004; ES = 1.80) than the SS. No other muscles, with the exception of BIC (−38%; *p* = 0.019; ES = 1.68)*,* showed a level of excitation significantly different by the SS. No coactivation indices were significantly different by the SS. The W1 synergy showed the activation of PEC, DOR, TRA, and BIC. The W2 synergy consisted in the activation of PEC, TRI, INF, DEL, and BIC. The W3 synergy showed the simultaneous activation of DOR and SER, while INF, TRA, and DEL were included in the W4 synergy.

## Discussion

The aim of the study was to evaluate the role of the different muscles during the execution of the SS element compared with the PSEs. The results confirmed the hypothesis and demonstrated that different muscle excitation, coactivation, and synergies characterize the various PSEs compared with the SS. In addition, the results common elements, such as the shoulder flexion, were detected in both SS and PSEs, while other elements were significantly different between SS and PSEs.

The BIC, DEL, and SER muscles seem to play a predominant role in the execution of the SS element on competition rings. The large excitation and synergistic activity of the BIC and DEL muscles in W2 synergy during the SS element are related to the strong shoulder flexion action required (22). In contrast, the excitation of PEC may compensate for the trunk instability because of the length of the ring's cables. A decrease in the strength of these muscles may result in a forward shift of the center of gravity, widening the rings and preventing the correct position of the SS. Although the primary action of PEC is to adduct the humerus, the INF, which is simultaneously activated in the W1 synergy, also stabilizes the shoulder and externally rotates the humerus (8). In addition, this muscle, in synergy with the DOR (both active in the W3 synergy), prevents the anterior slippage of the humeral head (which is depressed in the raised arm position) during the prone position adopted for perform the SS (1,24). The TRA and SER muscles (shown in W4 synergy) help to stabilize the scapula to maintain the proper position on rings. Similar muscle activation has been observed during the performance of the swallow exercise on competition rings (7). Both rings-support exercises (swallow and SS) require significant upper body strength to maintain the horizontal position of the body (19). The TRI muscle was not present in any of the 4 synergies extracted for the SS element. Consequently, this muscle may play a limited role in the execution of SS, such as keeping the forearm extended over the arm.

Comparisons between the SS and its 5 PSEs revealed many differences in activation, coactivation, and muscle synergies. With the exception of the DSP exercise, muscle excitation was lower in all PSEs compared with the SS element. This lower muscle excitation in PSEs may be explained by the characteristics of the exercises used by gymnasts to increase their strength. These exercises are not performed on the competition rings but rather using apparatuses that reduce the load and consequently the stress on the muscles and joints, while respecting some technical characteristics of elements performed on the competition rings.

This was observed in the SS_P_, where the reduced muscle activity detected in the PEC, TRI, TRA, and BIC muscles compared with the SS was attributed to the reduction in load represented by the body mass of the athletes and to the increased stability obtained by using a cable pulley system. This finding confirms that muscle excitation decreases when the load is reduced or when a stable condition is compared with an unstable condition (2,4). In addition, muscle coactivation between SER and TRA explains the higher excitation of SER and the lower excitation of TRA. The DEL/INF (20.1%) and BIC/TRI ratios (14.5%) were not significantly different and were very similar to those in the SS (19.9 and 9%, respectively). The W1 muscle synergy in the SS_P_ is the only synergy that included PEC and INF muscle. The other synergies included the same muscle activation of the SS element, with the exception of the BIC muscle's involvement in the W3 synergy. These results suggest that SS_P_ exercise is very similar to the main element executed on competition rings. However, BIC muscle activation differs from the SS in terms of excitation and muscle synergies. Therefore, the SS_P_ helps to replicate the SS body technique, emphasizing the position of the scapulae while reducing the involvement of the BIC muscle.

Similarly, the excitation of the muscles investigated in the recent study during the RPD exercise was significantly lower compared with SS, especially for the SER, TRA, and BIC muscles. This lower excitation is confirmed by the lower SER/TRA and the highest BIC/TRI coactivation indices compared with the SS element. These results suggest that, with the RPD exercise, athletes present a relevant abduction of their scapula at the end of the concentric phase, balancing the action of the load that pulls them up. In addition, and in contrast to the previous exercise, the muscle synergies of the RPD exercise were different from those of the SS, suggesting that arm adduction, rather than a shoulder flexion, occurred. Athletes may, therefore, use this exercise to strengthen the muscles connected to the scapula and humerus. To achieve a higher excitation in the other muscles, and in particular of the BIC muscle, additional general physical training may be needed.

The SS_SF_ is the only exercise that did not show significant differences in BIC muscle excitation (53.8%) compared with the SS. In addition, SER excitation was very high (79.8%), and TRA excitation was significantly lower (−74%), resulting in a significantly lower SER/TRA index (14.3%) compared with the SS. These results confirmed previous authors who reported a similar SER/TRA ratio (16%) in high plank exercise (10). In the SS_SF_, the BIC/TRI ratio is the closest to the SS value. However, the SS_SF_ shows different muscle synergies activation for BIC and INF. Thus, given the muscular excitation and the coactivation indices, the SS_SF_ may promote strength gains of the serratus, biceps, and deltoid muscles.

The DSD exercise was characterized by very low muscle excitations registered in PEC, TRI, TRA, and BIC. Despite the significantly lower excitation of the PEC muscle in DSD compared with SS, the first showed the highest PEC excitation among all PSEs. The analysis of muscle synergies revealed that in the DSD exercise, the synergistic activation of the shoulder muscles seems to be modified, with a reduced contribution of the BIC (W3 synergy shows the lowest factor of BIC involvement among all synergies in each exercise). Specifically, in this exercise, PEC and BIC engage in synergy for arm adduction (14). As a result, the DSD action is more likely to strengthen the PEC muscle and focus on arm adduction rather than on shoulder flexion, element that typically characterizes the SS element.

In the DSP exercise, muscle excitation differed from SS for PEC and BIC muscle activation only, while no statistical differences were detected for coactivation indices. The BIC/TRI ratio (12.4%) was similar to that of the SS (9%), whereas the PEC/DOR ratio was higher (21.4%) than the SS (9.9%). The analysis of muscle synergies revealed different simultaneous muscle activations for each W synergy. Although DSP W2 involved a greater number of muscles than SS W2 (including the PEC), it was the only exercise among those studied that included both the BIC and DEL muscles, similar to SS. These findings, together with the high excitation of the DEL (102% of SS), suggested that these muscles have a key role in DSP to perform a correct shoulder flexion. Our results align to the previous EMG analysis of front raises exercise, in which the DEL and PEC (clavicular head) contributed to shoulder flexion (12). In addition, the DSP exercise induced a lower excitation of the INF muscle during the DOR and PEC actions compared with the SS.

In conclusion, this is the first study to describe the principal muscular activity during the SS element and its related PSEs. The SS on competitive rings is performed using simultaneous actions of the BIC and DEL muscles, while the other muscles contribute more synergistically to stabilize the athlete's shoulder joint. The analysis of muscle excitations, coactivation indices, and synergies between active muscles revealed different uses of the PSEs during gymnastics training routine. However, a limitation of this study was the inability to quantify the load of the isometric hold position during the execution of the RPD exercise because of the variability in the resistance produced by the elastic bands. As a result, although the number of repetitions performed by the athletes is known, the precise force produced could not be determined. In addition, the study did not account for how different loads may affect muscle excitation, coactivation, and synergies in both the SS and the PSEs.

**Practical Applications**

The results of this study support the inclusion of the PSEs in the training routine of gymnasts to stimulate different muscle actions relevant to the SS. The SS_P_ is considered suitable for the early phases of training, emphasizing the SS technique. Conversely, the RPD exercise is more proper for strengthening the stabilizing muscles of the scapula and, together with the DSD exercise, can be effectively used to target the muscles involved in the humeral adduction. Given the muscular action of the BIC and the TRA during these 2 exercises, it may be recommended to enhance the strength of these muscles when RPD and DSD exercises are incorporated into the training routine. The SS_SF_ exercise may be adopted to increase strength of the SER, the DEL, and the BIC muscles, while the DSP exercise may be performed to strengthen the shoulder flexion action.
